# Multifunctional biofunctionalized hybrid nanoantifungals with novel active coating agents based on cinnamaldehyde- and β-cyclocitral-modified polydopamine

**DOI:** 10.1016/j.mtbio.2025.102645

**Published:** 2025-12-10

**Authors:** Maria Paz García-Simarro, Maria Mondéjar-López, Joaquin C. García-Martínez, Antonio Cuesta-Casas, Sergio Casas-Tintó, Oussama Ahrazem, Lourdes Gómez-Gómez, Enrique Niza

**Affiliations:** a Instituto Botánico. Departamento de Ciencia y Tecnología Agroforestal y Genética. Universidad de Castilla-La Mancha, Campus Universitario s/n, 02071 Albacete, Spain; b Escuela Técnica Superior de ingenieros Agrónomos y Montes. Departamento de Ciencia y Tecnología Agroforestal y Genética. Universidad de Castilla-La Mancha, Campus Universitario s/n, 02071 Albacete, Spain; c Universidad de Castilla-La Mancha, Departamento de Química Inorgánica, Orgánica y Bioquímica, Facultad de Farmacia, C/ José María Sánchez Ibáñez s/n, 02008, Albacete, Spain; d Universidad de Castilla-La Mancha, Regional Center for Biomedical Research (CRIB), C/ Almansa 13, 02008, Albacete, Spain; e Institute for Rare Diseases Research, Instituto de Salud Carlos III (ISCIII), 28222 Madrid, Spain

**Keywords:** Advanced nanomaterials, Antifungal, Terpenoids, pH-responsive release, Crop protection, Sustainable materials

## Abstract

Fungal pathogens pose serious threats to global agriculture, causing substantial crop loss and food security concerns. Current solutions often rely on conventional pesticides, generating negative environmental impacts. This study presents the development of two novel multifunctional hybrid nanoparticles designed to provide a sustainable alternative for crop protection and growth promotion. These nanoparticles are based on dendritic mesoporous silica nanoparticles (dMSNs) and functionalized with innovative coating agents derived from cinnamaldehyde- (CIN) and β-cyclocitral (βETA) -modified polydopamine (DOPA). The purpose of this research was to create a system that could deliver antifungal agents and support plant development simultaneously, especially under pathogen-induced stress. Nanoparticles exhibited a dual-release mechanism with pH-responsive kinetics, releasing up to 94 % of geraniol (GER) and 81 % of compound βETA, at pH 5. This confirms their ability for targeted, stimulus-triggered delivery. In vitro tests showed strong antifungal activity against several plant pathogenic fungi, with minimum inhibitory concentrations down to 0.078 mg/mL. Microscopic analysis revealed significant disruption of fungal mycelia after treatment, confirming the antifungal mechanism. In vivo biosafety was established through assays on *Drosophila melanogaster.* Furthermore, experiments on plants infected with *Fusarium oxysporum* demonstrated enhanced seed germination and early plant development. Treated plants showed improved root and shoot growth, higher chlorophyll content, and restored levels of key physiological markers like polyphenols and carotenoids. This study reports two dual-functional nanoparticles that improve the control of fungal pathogens while simultaneously promoting early plant development. The findings highlight their potential as sustainable nanobiotechnological tools for protecting crops and enhancing productivity in agricultural systems affected by fungal diseases.

## Highlights

• Cinnamaldehyde- and β-cyclocitral-modified polydopamine coatings on dMSNs create multifunctional antifungal nanoformulations.
• pH-responsive dual release: up to 94 % geraniol and 81 % β-cyclocitral released at pH 5.
• Antifungal efficacy: minimum inhibitory concentrations (MICs) as low as 0.078 mg mL^−1^, with marked mycelial disruption.
• Seed nanopriming in wheat: restored germination/seedling emergence and metabolites under *F. oxysporum* challenge.
• *In vivo* biosafety: no significant toxicity in the *Drosophila melanogaster* model.

## Graphical abstract

**Figure:**
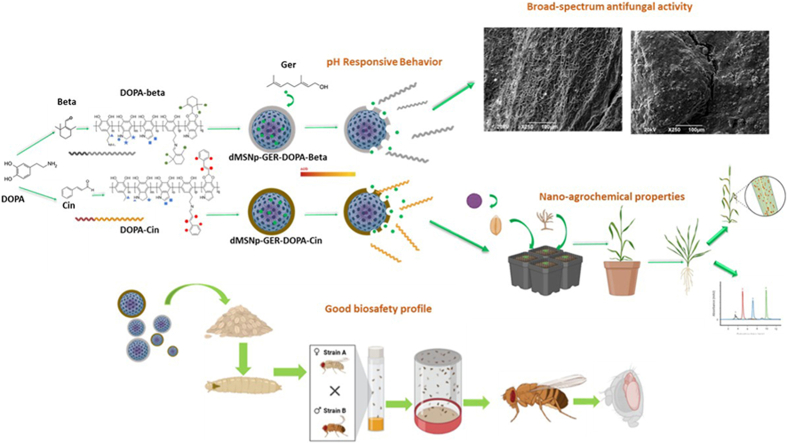

## 1 Introduction

Fungal crop diseases are the leading cause of crop failure worldwide, with 85 % of pathogenic crop diseases destroying 20–40 % of the annual global crop, resulting in economic losses of 600-4 billion per year. [1,2]. To mitigate these losses approximately 400 thousand tons of fungicides (20 % of the total pesticide), are annually used to control the main phytopathogens enhancing the crop yields [3]. However, the unspecific targeting, the non-controlled release and their application process inadvertently leads to excessive use in seed dressing and foliar application. Notably, the widespread use of pesticides causes significant negative effects on environment and human health, including inhibiting the growth of nontarget organisms, disrupting the nutritional quality of crop grains, and the risk of exposure and toxicity to humans [4]. This situation has led to the establishment of different regulatory frameworks in the USA and the EU, such as the “green agreement”, with the aim of reducing pesticide uses by up to 50 % by 2023 and, ultimately, banning most chemically synthesized pesticides, such as triazols [5].

Nanotechnology offers an efficient approach to enhance plant health and improve crop management thanks to the unique physicochemical properties of nanomaterials [6]. The integration of nanotechnology into agricultural practices has given rise to NanoAgri, an interdisciplinary field focused on developing and applying diverse nanomaterials and nanoformulations. These tools—such as nanobiosensors, nanofertilizers, nanopesticides, and nanocarriers—are engineered to enhance the precision and efficiency of crop management while minimizing collateral impacts on non-target organisms, contributing to more sustainable agricultural systems [7,8].

A central thrust of this field is the encapsulation of bioactive natural products, including essential oils, plant extracts, and terpenoid metabolites, within nanostructured carriers. This strategy underpins a new generation of green nanoagrochemicals that can serve as eco-compatible complements or alternatives to conventional synthetic pesticides. By actives and enabling controlled, targeted release, nanoencapsulation improves stability, solubility, and bioavailability, enhancing agronomic performance and facilitating alignment with evolving regulatory frameworks [9].

Terpenes, in particular, are constrained by poor aqueous solubility, high volatility, and rapid field degradation; nanocarrier design can mitigate these liabilities. For carvacrol, eugenol, and cinnamaldehyde, nanoformulations have demonstrated greater antifungal potency, prolonged residual activity, and improved integration into integrated pest management (IPM), with downstream benefits for yield and post-harvest quality [[10], [11], [12]].

In parallel, bio-based antimicrobial materials, for example, matrices of carboxymethyl chitosan or polylysine combined with bioactive molecules, are expanding activity spectra and suppressing biofilm formation, opening additional routes to embed nano-enabled functions into crop protection programs [7].

Dendritic silica mesoporous nanoparticles (dMSNs) are a new generation of porous materials that have gained great attention compared to other mesoporous silicas due to attractive properties, including straightforward synthesis methods, modular surface chemistry, high surface area, tunable pore size, chemical inertness, particle size distribution, excellent biocompatibility, biodegradability, and high pore volume compared with conventional mesoporous materials [7]. In addition, easy modification of the surface of dMSNs with different molecules such as polymers may allow obtaining a stimuli-responsive behavior, achieving a more specific delivery of the encapsulated compounds. Polydopamine is a biomimetic self-adherent polymer, which can be easily deposited on a wide variety of materials such as dMSNS conferring a different stimuli responsive behavior such as pH and NIR responsive capacity, high photothermal conversion efficiency and strong bioadhesion to many surfaces [8].

In this study, we developed two novel dual-functional hybrid nanomaterials based on dMSNs encapsulating GER, and coated with a newly synthesized biopolymer composed of polydopamine covalently functionalized with either CIN or βETA with the aim to develop a new nano-antifungal as seed dressing agent in wheat seeds These nanomaterials demonstrate: (1) pH-responsive release behavior, enabling controlled delivery of both the surface-bound and internally encapsulated terpenes; (2) a dual antifungal mechanism of action; (3) broad-spectrum antifungal activity against major phytopathogenic fungi; (4) high efficacy as seed treatment agents, enhancing germination rates and improving physiological and defense-related parameters in treated seeds; and (5) a favorable biosafety profile validated through in vivo assays. Together, these findings highlight the potential of these two advanced nanoparticles as environmentally sustainable and targeted alternatives for fungal disease management in agriculture.

## 2 Material and methods

### 2.1 Materials

Cetyltrimethylammonium tosylate (CTATos), triethanolamine (TEAH3), 1-butyl-3-methylimidazolium trifluoromethanesulfonate (BMIM), tetraethyl orthosilicate (TEOS), chitosan (CH), dopamine hydrochloride (DOPA), pluronic (F127) and solvents were purchased from Sigma-Aldrich (Madrid, Spain) and GER was purchased from Pranarom (Barcelona, Spain). The organisms used for the antifungal assays consisted of the species of *Fusarium oxysporum*, *Aspergillus niger* and *Penicillium citrinum*, which were isolated in previous studies [9].

### 2.2 Synthesis of dMSNs

Preparation of dendritic mesoporous silica nanoparticles (dMSNs) were prepared according to the sol-gel method described by Ref. [51] with minor modifications. Briefly, 2.24 g of F127 was dissolved in 100 mL of deionized water and heated to 80 °C in an oil bath while stirring at 600 rpm. After complete solubilization of the surfactant, 1.92 g CTATos, 0.21 g TEAH3 and 0.02 g BMIM were added to the solution, which became clear and foamy, and the mixture was stirred at 600 rpm and 80 °C for 1 h. At this point, 14.48 g of TEOS were added and the mixture was stirred for another 2 h at the same temperature and stirring conditions to form a white suspension. Finally, the suspension was centrifuged and washed 2 times with EtOH and 2 times with water, after which the precipitate was dried in an oven at 80 °C overnight.

### 2.3 Geraniol encapsulation into dMSNs (dMSNs-GER)

The GER encapsulated dMSNs were obtained by adding 50 mg of dMSNs to 0.2 mL of GER and leaving the mixture in agitation overnight. They were then washed 3 times with EtOH and lyophilized. The amount of GER loaded on dMSNs-GE was quantified by measuring the absorbance of the supernatant at 202 nm.

### 2.4 Coating of dMSNs with polydopamine

The dMSNs-DOPA was obtained by adding 50 mg of dMSNs ot dMSNs-GER to a dopamine solution of 0.5 mg/mL of TRIS buffer at pH 8 and leaving it in agitation overnight giving rise to a brown-black suspension. After that, it was centrifuged for 10 min at 13000 rpm and washed twice with water. The supernatant after washing was frozen until further use. Finally, the precipitate was lyophilized.

### *2.5* *Preparation of dMSNs coated with CIN and* βETA *modified polydopamine*

The dMSNs-DOPA = Cin and dMSNs-DOPA = βETA were obtained by adding 50 mg dMSNs or dMSNs-GER to a dopamine solution of 0.5 mg/mL of TRIS buffer at pH 8 previously homogeneized with of Cin or βETA at 2,5 mg/mL, the suspensions were keeping overnight under agitation givin rise to a brown suspension (dMSNs-DOPA = Cin) and grey suspension (dMSNs-DOPA = βETA). After that, the nanoparticles were collected after centrifuged for 10 min at 13000 rpm and washed twice with water. The supernatant after washing was frozen until further use. Finally, the precipitate was lyophilized.

### 2.6 Determination of the encapsulation and loading efficiency of GER

A sample of the aforementioned supernatants (1 μL for dMSNs-GER and 100 μL coated dMSNs) was diluted with ethanol and then analyzed by UV-vis spectrophotometry over a wavelength range of 190–400 nm, covering the maximum absorption wavelength of Geraniol (202 nm)

The loading capacity (LC) and encapsulation efficiency (EE) of the GER were calculated as follows [10]:

(1) LC % = (weight of encapsulated GER (mg)) / (weight of total (GER encapsulated (mg)) × 100 %

(2) EE % = (weight of encapsulated GER (mg)) / (weight of GER feeding (mg)) × 100 %

### 2.7 Determination of the β-cyclocitral and cinnamaldehyde grafting efficiency

A sample of dMSNs-DOPA = Cin and dMSNs-DOPA = βETA supernatants (100 μL) was diluted with ethanol and then analyzed by UV-vis spectrophotometry over a wavelength range of 190–800 nm, covering the maximum absorption wavelength of βETA (255 nm) and Cin (220 nm and 290 nm). Moreover, these values were confirmed with FT-IR and NMR spectroscopy. The grafting efficiency of terpene and apocarotenoid to DOPA were calculated as follow:

GE % = (weight of grafted CIN or βETA (mg)) / (weight of initial CIN or βETA (mg)) × 100 %

### 2.8 Instrumental characterization of nano-materials

#### *2.8.1* *UV-Vis spectrum of dMSNs-DOPA = CIN and dMSNs-DOPA =*βETA

UV–vis spectroscopy was applied to evaluate the incorporation of CIN and βETA in a polydopamine coated nanoparticles after the polymerization reaction, using UV-1900i Shimadzu. The wavelength range went from 200 nm to 800 nm when observing characteristic peaks corresponding to CIN and βETA presence. Briefly, dMSNs-DOPA, dMSNs-DOPA = CIN and dMSNs-DOPA = βETA were suspended in distilled water at low concentration (0,1 mg/mL) to ensure the unsaturation of the absorbance to facilitate the spectral analysis.

#### 2.8.2 ATR-FTIR studies

IR spectra was recorded on an attenuated total reflectance-Fourier transform infrared (ATR–FTIR) spectrophotometer (VARIAN 640-IR with a Pike Diamond/KRS-5 HS Performance Crystal Plate) and the main peaks were given in cm−1. ATR allows us to use the samples directly in solid or liquid state without the need of KBr or lugol's iodine matrix. Specifically, for NPCH and GEO-NPCH, 20 mg nanoparticles were powdered in a mortar, the thin solid was placed on the diamond plate and pressed until a homogeneous pellet was obtained. GEO, being liquid, a drop of approximately 200 μL was placed on the plate and the tip was placed in such a way that the surface tension of the drop covered the diamond plate homogeneously. 256 scans were acquired at an instrument resolution of 1 cm−1 over the spectral range between 650 and 4000 cm−1 owing to the frequency cutoff of the ATR–FTIR internal reflection element (IRE) used**.**

#### 2.8.3 Thermal and thermogravimetric studies

The thermal decomposition mechanisms were determined on a thermogravimetric analyser (TGA Q20, TA Instruments) fitted with a standard platinum pan. The differential scanning calorimetry (DSC) experiments were carried out using a DSC Q50 system (TA Instruments) equipped with a standard aluminium pan with 10 °C/min increasing heat rate (30–320 °C) to investigate the thermal stability of pure GEO, NPCH and GEO-NPCH. A sample of indium was used as reference. In all cases, samples of about 3 mg were heated at a 10 °C/min rate under nitrogen atmosphere.

#### 2.8.4 The particle characterization of the nano-formulations

Size, zeta potential and polydispersity index (PDI)) was determined by Dynamic Light Scattering (DLS) using a Zetasizer (3000HSM Malvern Ltd., IESMAT, Spain) with the following specifications: silica refractive index (IR) of 1.460, absorption index of 0.010 and water solvent RI: 1.33, and viscosity of 0.8872 cP. Measurements were performed in triplicate.

#### 2.8.5 Transmission electron microscopy studies

Ultrastructural analyses were performed in a JEOL-1400Flash electron microscope equipped with a digital high-sensitivity sCMOS camera (Jeol Ltd., Tokyo, Japan). Digitized electron images were modified for brightness and contrast by using Adobe Photoshop CS5 (Mountain View, CA).

### 2.9 In vitro GER release of nanoparticles

To determine the release profile of GER from the nanoparticles, 5 mg of dMSNs-GER, dMSNs-GER-DOPA = Cin or dMSNs-GER-DOPA = βETA were added to dialysis membranes placed in 20 mL of PBS at pH 7 and pH 5.4 under continuous stirring at 150 rpm to ensure homogeneity of the medium. The amount of released GER was estimated by measuring the absorbance at 202 nm at different times in triplicates.

The release kinetics of GER from the proposed nanoparticles were assessed using four commonly used mathematical models: Zero-order, First-order, Higuchi-order, and Korsmeyer– Peppas. Equations used in the mathematical models to determine the GER release parameters are as follows:

(3) Qt = K0. t Zero-order model

(4) ln(100 − Qt) = ln100 − K1. t First-order model

(5) Qt = KH.t1/2 Higuchi model

(6) Qt = KKP.tn Korsmeyer-Peppas model

### *2.10* *In vitro release of CIN and* βETA *from coated nanoparticles*

To determine the release profile of CIN and βETA from the nanoparticles, 5 mg of dMSNs-GER-DOPA = Cin and dMSNs-GER-DOPA = βETA were added to dialysis membranes placed in 20 mL of PBS at pH 7 and pH 5.4 under continuous stirring at 150 rpm to ensure homogeneity of the medium. The amount of released CIN and βETA was estimated by measuring the absorbance of βETA (255 nm) and Cin (220 nm and 290 nm) at different times in triplicates.

The release kinetics of CIN and βETA from the proposed nanoparticles were assessed using four commonly afomentioned equations.

### 2.11 Antioxidant properties of nanoparticles

FRS, free radical scavenging activity, was determined as described previously [5]. Briefly, 0.5 mL for each concentration (1 mg/mL, 500 μg/mL, 250 μg/mL and 125 μg/mL) of synthetized nanoparticles was mixed with 0.1 mM ethanolic DPPH radical solution (1.5 mL) and then mixed and kept in the dark at room temperature for 30 min. The absorbance of the solution was measured at 517 nm. The FRS was calculated by % = (A0 − A1/A0) × 100. Where A0 is the absorbance at zero time and A1 is the absorbance after 30 min of incubation.

### 2.12 Antifungal evaluation of multifunctional nanoparticles

#### 2.12.1 In vitro antifungal assay

The antimicrobial activity and minimum inhibitory concentration (MIC) of unencapsulated GER and the proposed nanocarriers were tested against the most common pathogenic microorganisms affecting in different crops. The antimicrobial activity of the nanoparticles against *F. oxysporum, A. niger and P. citrinum*. was tested using the broth microdilution method [9,11]. Stock cultures were prepared from Culti-Loops ™ (Sigma-Aldrich, Madrid, Spain) in Potato Dextrose Broth (PDB) at 37 °C. Standardized inoculum was then created by dilution in Müller–Hinton medium to a final density of 0.5 McFarland units by densitometer McFarland type DEN-1B (Biosan, Riga, Latvia). GER, dMSNs-GER-DOPA, dMSNs-GER-DOPA = Cin, dMSNs-GER-DOPA = βETA and their empty nanoparticles were tested at different concentrations from 5 mg/mL to 0.05 mg/mL applying the microdilution process described in Ref. [12]. Tebuconazole was used as the standard at fungicidal dose to ensure the activity (5 mg/mL). After treatment, the plates were incubated for 48 h at 30 °C for fungi and all were treated with 10 μL of 3-(4 5-dimethylthiazol-2-yl)-2 5-diphenyltetrazolium bromide (MTT; 5 mg/mL in PBS; Sigma). Finally, plates were incubated overnight at room temperature, followed by the addition of 100 μL of MTT solvent (0,1 NHCl in anhydrous isopropyl alcohol) [13]. The experiment was carried out in triplicate.

#### 2.12.2 Antifungal examination by Scannig electron microscopy

To observe the effect of the obtained nanoparticles on the fungal morphology of *F. oxysporum* hyphae, mycelia cultured in PDB medium for 2 days were treated with dMSNs-GER-DOPA = Cin and dMSNs-GER-DOPA = βETA separatly with MIC values for each treatment (0.3 mg/mL for dMSNs-GER-DOPA = Cin and 0.4 mg/mL dMSNs-GER-DOPA = βETA) for 24 h [14]. Then, the mycelia were collected and were sputtered with Pt and observed with a Jeol 7800 F electron microscope at 20 KV.

#### 2.12.3 Determination of lipid peroxidation

MDA content was measured using a 2-thiobarbituric acid reaction method. 0.5 g of fresh mycelium was homogenized in 5 mL of 5 % (W/V) trichloroacetic acid and the homogenate was centrifuged at 10,000 g for 15 min at room temperature. The supernatant was mixed with an equal volume of 2-thiobarbituric acid (0.67 % in 20 % [w/v] trichloroacetic acid) and the mixture was boiled for 30 min at 100 °C, followed by centrifugation for 10 min at 7500 g to clarify the solution [15].

### 2.13 Effects of the nanoparticles as nanopriming agents in wheat seeds

#### 2.13.1 Antioxidant properties of nanopriming agents in wheat seeds

In order to evaluate and related the antioxidant properties of nanoparticles with the antioxidant effect in treated seeds we examinate the germination rate under presence of higher concentration of oxidizing agents KMnO4 (0.2 %) and H_2_O_2_ (10 %). Briefly, 100 g batches of wheat (*Triticum vulgare*) seeds were divided into the following groups: Control (Non treated seeds), dMSNs-GER-DOPA = Cin and dMSNs-GER-DOPA = βETA. For the nanotreated, seeds were soaked in the respective treatment at 5 mg/mL of each nanoparticle to ensure a successful seed dressing and then dried at room temperature. Then, the seed batches were soaked in KMnO_4_ at 0.2 % and H_2_O_2_ at 10 % during 20 min under vigorous stirring [16]. After treatment, seeds were dried at room temperature. Batches of 100 seeds of each cereal were placed in sterile wet filter paper and incubated at 25 °C for 5–7 days in a germination chamber with 21.8 °C, short day length with 8 h light and 16 h dark.

#### 2.13.2 Fungi-inoculated germination assay

To assess the efficiency of new nanoparticles as effective seed nanopriming agents, we examined the germination rate post-treatment. In brief, four 200 g batches of wheat seeds were divided into the following groups: control (+) (seeds neither inoculated with fungi nor treated), F.ox-UT as the inoculated control (seeds inoculated with fungi but not treated (named as control infected)), dMSNs-GER-DOPA = Cin (seeds inoculated with fungi and treated), and dMSNs-GER-DOPA = βETA (seeds inoculated with fungi and treated). For the nano-treatment, take in account the antioxidant concentration of both multifunctional nanoparticles, 200 g of seeds were soaked in the respective treatment at 5 mg/mL of each nanoparticle to ensure a successful seed dressing and then dried at room temperature. The wheat seed batches were placed in individual pots for each treatment. Seeds inoculated with fungi were treated by adding 1 ml of *F. oxysporum* suspensions with 3000 spores/ml to the surface of the seeds to ensure adequate inoculation and placed in soil to improve seed adhesion and spore dispersion through the soil following the method described in Refs. [11,17,18]. The impact on seed germination was assessed by counting the number of surviving seeds. The experiment was conducted in triplicate. The plant development study was carried out during 30 days and the surviving plants were collected for physiological and response analysis. The experiment utilized a completely randomized design with three independent biological replicates (n = 3) per treatment group. This replicate structure involved randomizing the placement of pots to mitigate the effects of spatial environmental variation.

#### 2.13.3 Damage index

Disease evaluation in the surviving plants was conducted based on the incidence (percentage of affected plants) and the severity of symptoms (chlorosis, wilting, necrosis, defoliation), using a scale from 0 to 4 (0 = healthy plant or no symptoms; 1 = plant affected 1–33 %; 2 = 34–66 %; 3 = 67–99 %; 4 = dead plant). Subsequently, the percentage disease progress curve was calculated.

#### 2.13.4 Effects of the different nanoparticles on wheat plant physiology and response parameters

To evaluate the biosafety or phytotoxicity effects of these novel nanopriming treatments, the following metabolites involved in proper plant metabolism, development and stress response were evaluated in the surviving plants.

#### 2.13.5 Determination of chlorophyll content

The total chlorophyll content in the leaves of treated seed plants was assessed as in Ref. [19] with some modifications. Specifically, 25 mg of the powdered leaves were extracted using 2 mL of 100 % acetone. The samples were then centrifuged at 10,000 g for 10 min and the absorbance of the supernatant was measured at 662 and 644 nm.

#### 2.13.6 Determination of total flavonoid content

The Flavonoid content in the aqueous extract was determined by a colorimetric method using AlCl3∗6H2O [20]. Briefly, 0.250 mL of the aqueous extract was mixed with 0.75 mL of ethanol, 0.05 mL of 10 % AlCl3∗6H2O, 0.05 mL of 1 M CH3COOK and 1.4 mL of H2O. After mixing, the color change was evaluated at 415 nm. Quercetin (QE) was used as a standard at different concentrations (8–500 ppm).

#### 2.13.7 Determination of total polyphenols

To determine the total amount of polyphenols in the aqueous extract, the Folin-Ciocalteau method was used [20]. Briefly, 0.1 mL of aqueous extract was mixed with 2 mL of 2 % Na2CO3, 2.8 mL of H2O and 0.1 mL of Folin-Ciocalteau reagent. After mixing, the color change was measured using absorbance at 750 nm. Gallic acid (GAE) was used as a standard at different concentrations (10–200 ppm).

#### 2.13.8 Determination of carotenoids

Carotenoids were quantified as in Ref. [21] with some modifications: 25 mg of powdered leaves were extracted with 2 mL of 100 % acetone. The samples were then centrifuged at 10,000 g for 10 min and the absorbance of the supernatant was measured at 450 nm.

### 2.14 In vivo toxicity evaluation of nanoparticles

#### 2.14.1 Drosophila stocks

Fly stocks were maintained at 25 °C, 60 % humidity and 12/12 h light/dark cycles. Fly stocks used were UAS-LacZ (BL8529), UAS-myrRFP (BL7119), repo-Gal4 (BL7415), from the Bloomington Stock Center (https://flystocks.bio.indiana.edu/).

#### 2.14.2 Nanoparticle toxicity evaluation

Toxicity evaluation of dMSNs-GER = DOPA = CIN and dMSNs-GER = DOPA = βETA were carried out using *Drosophila* in vivo models. The nanoparticles were administered to *Drosophila* embryo and larvae through the diet mixed with the food. Nanoparticles were suspended in water to the specific final MIC values of each one.

The diluted nanoparticles were mixed into standard food heated at 56 °C, a temperature at which it remains in liquid form, enabling the mixture of the compound with the food. After 10 days, we evaluated the number of individuals that survived and reached adulthood and percent of the progeny.

#### 2.14.3 Drosophila Larval brain dissection and immunostaining

We dissected 3rd instar larvae in phosphate buffered saline (PBS). We held the head of the larva with tweezers, and we inserted forceps through the opening of the section. Next, we pushed until the internal wall and viscera were exposed to the exterior. Finally, the viscera of the larva were removed, and the brain was located and separated from the rest of the body.

We fixed *Drosophila* tissues with 4 % formaldehyde (PFA) for 20 min at room temperature (RT). Next, we washed the samples three times in PBS 1x + 0.3 % Triton X-100 (PBT) for 10 min under stirring at RT. Next, we blocked the samples in (0.3 % PBT + 5 % BSA) under agitation for 30 min at RT. We incubated the primary antibody in blocking solution at 4 °C overnight. Next, we washed the samples in PBT (3 × 15 min) and we incubated the secondary antibody (fluorochrome-conjugated) in blocking solution for 2 h in the dark and under constant agitation at RT. Finally, we washed the samples in PBT (3 × 15 min) and we mounted the brains on microscope slides, using mounting medium (Vectashield with DAPI), and covering the slide with a coverslip until analysis.

We used the primary antibody anti-Repo (DSHB, 1/100), which recognizes the transcription factor encoded by the *repo* gene, to mark the nuclei of glial cells.

Secondary antibodies (Thermofisher): anti-mouse Alexa 488. DNA was stained with 2-(4-amidinophenyl)-1H-indole-6-carboxami-Dine (DAPI) at 1 μM in Vectashield mounting media (Vector Laboratories).

### 2.15 Statistics

The obtained data were statistically analyzed using one-way ANOVA and Dunnet's Multiple Comparisons test with the statistical software GraphPad Prism version 5.0.0 for Windows, GraphPad Software, San Diego, California USA. The differences were tested on < 0.05 (95 % probability level).

## 3 Results and discussions

### 3.1 Synthesis and characterization of nanomaterials

**Fig. 1:**
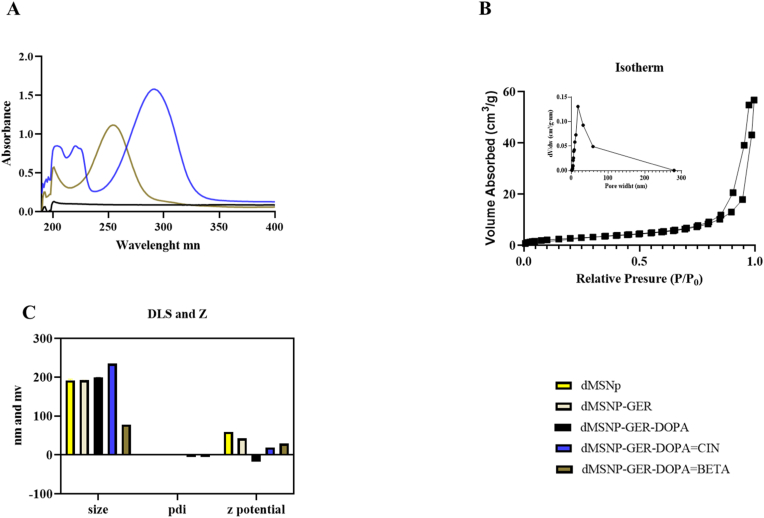
A) UV-vis spectra of dMSNs-DOPA, dMSNs-DOPA = CIN and dMSNs-DOPA = βETA. B) N2 adsorption-desorption isotherms of dMSNs. C) DLS and Z potential measures of different obtained nanoparticles. Data was generated as a result of three independent experiments.

The dendritic silica nanoparticles were obtained following a size controllable method using imidazolium ionic liquids (ILs) and Pluronic F-127 as surfactant [22,23]. The hydrodynamic diameter of the unloaded dMSNs was measured at 193 ± 2 nm, exhibiting a narrow size distribution and a low polydispersity index (PDI), indicative of uniform nanoparticle dispersion (Fig. 1). Notably, the empty dMSNs displayed a strongly positive surface charge, with a zeta potential of +59.1 mV. This high cationic surface potential is likely attributable to the presence of amine functionalities introduced via the BMIM-based ionic liquid and alkylamine surface modifications. Furthermore, this value confirms the high stability of dMSNs in aqueous suspensions, being higher than +30 mV [24]. After terpene encapsulation, the dMSNs-GER decreased their zeta potential reaching a surface charge of +42 mV, still exhibiting high stability and cationic charge properties. The active and dual coating of the nanoparticles was carried out by the reaction between dopamine monomers with Cinnamaldehyde (dMSNs-DOPA = Cin) and β-cyclocitral (dMSNs-DOPA = βETA) through covalent bond formation and subsequent oxidative self-polymerization. The results showed in Fig. 1 confirm a successful coating of DOPA = TERPENES displayed different average diameters and zeta potential values respect uncoated nanoparticles achieving 199 ± 1 nm in the case of dMSNs-GER-DOPA 235 ± 0 nm for dMSNs-GER-DOPA = Cin and 78 ± 0 nm for dMSNs-DOPA = βETA. The reduced average size observed for the βETA -grafted polydopamine-silica nanomaterials is likely attributable to enhanced colloidal stability, which prevents nanoparticle aggregation and promotes Brownian motion, thereby leading to lower hydrodynamic diameters as measured by dynamic light scattering (DLS) [25]. Furthermore, the novel βETA -based coating did not induce an increase in particle size heterogeneity or destabilization of the suspension, as evidenced by the narrow size distribution and PDI values comparable to those of uncoated dMSNs (Fig. 3 c).

**Fig. 2:**
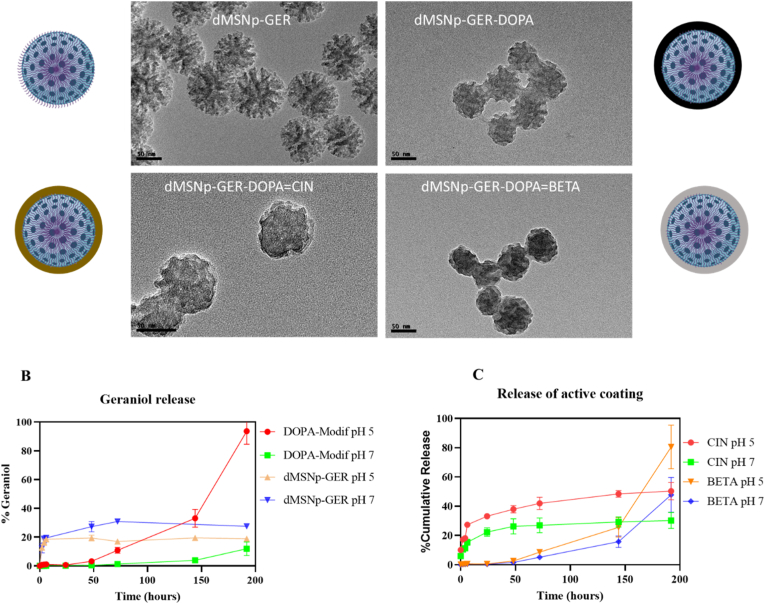
A) TEM images of uncoated dMSNs-GER and surface modified nanoparticles with proposed coatings. Release profile of Geraniol (B) and cinnamaldehyde and βetacyclocitral (C) from modified nanoparticles. Data was generated as a result of three independent experiments.

**Fig. 3:**
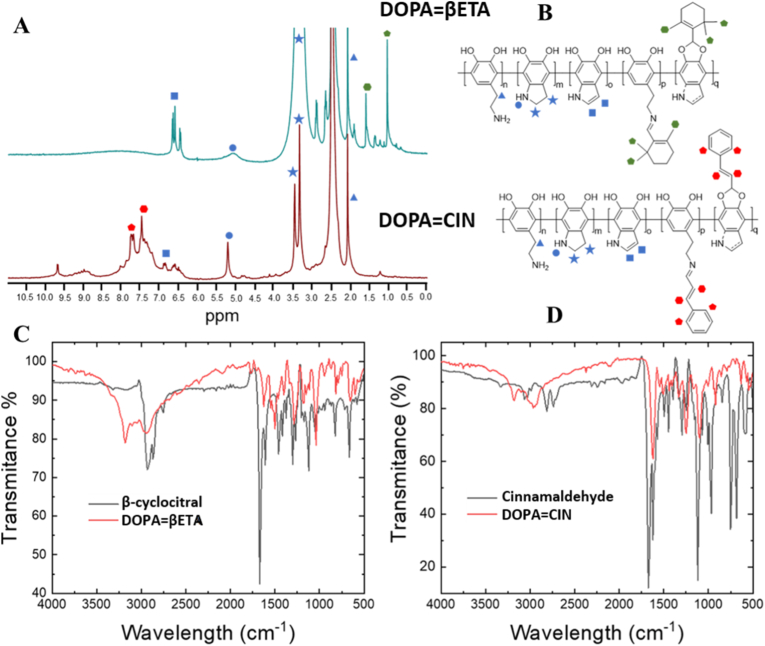
A) ^1^H-RMN spectra of DOPA = βETA and DOPA = CIN. B) possible structures of both substituted polymers. C and D) FTIR spectra of both polymers and the corresponding terpenene.

The successful deposition of the polydopamine (DOPA) coating was confirmed by a reversal of the surface charge of the silica nanomaterials, from strongly positive to negative, reaching values of −17 ± 0 mV. This behavior is consistent with previous reports and is attributed to the deprotonation of DOPA amine groups at neutral pH [26]. Remarkably, the two newly developed nanomaterials exhibited a shift back toward positive surface potentials compared to ungrafted polydopamine. Specifically, the zeta potentials were +19 ± 0 mV for dMSNs-GER-DOPA = CIN and +29 ± 0 mV for dMSNs-GER-DOPA = βETA, suggesting that the covalent attachment of CIN or βETA introduced additional amine functionalities that contributed to the increased surface charge. Furthermore, the high surface charge of dMSNs-GER-DOPA = βETA (approximately +30 mV) supports its excellent colloidal stability and the formation of a homogeneously dispersed suspension.

As shown in Fig. 2A, transmission electron microscopy (TEM) images reveal that the synthesized dMSNs exhibit a well-defined dendritic morphology, with an average diameter of approximately 80 nm and a homogeneous population. The characteristic radial pore architecture, attributable to the presence of multichannel structures, is clearly visible. These findings are consistent with those reported by Deng et al., who synthesized similar dendritic silica nanostructures via a sol-gel method, obtaining average sizes close to 100 nm [[27], [28], [29]].

Subsequent TEM analysis of the biopolymer functionalized nanomaterials further confirmed the successful surface coating of the dMSNs. The presence of a darker outer layer surrounding the nanoparticles was evident, corresponding to the polydopamine-based coating. This coating partially obscured the multichannel structure and blurred the dendritic features, resulting in a more spherical overall morphology. Notably, in the βETA -functionalized nanoparticles, an increase in electron density was observed, indicative of efficient and uniform surface functionalization. These morphological transformations and the thickening of the outer coating layer are in agreement with previous studies, such as that of Xiujuan et al., in which star-shaped silica nanoparticles exhibited a similar loss of structural definition following DOPA coating, further supporting the effectiveness and reproducibility of the functionalization strategy [8].

Successful grafting of β-cyclocitral and cinnamaldehyde onto polydopamine was confirmed by FT-IR, ^1^H NMR and Uv-vis spectroscopy. The structure of dopamine polymer is very complex, and different forms of the monomers that constitute it have been described, although the majority are those shown in Fig. 3 [30]). On the contrary, the structure of β-cyclocitral and cinnamaldehyde is well known and very different from each other, which will allow us to easily identify their incorporation into polydopamine by NMR. While cinnamaldehyde is completely aromatic, β-cyclocitral is not aromatic, which means that each essential oil will group its signals in different parts of the NMR spectrum. Similarly, we can analyze the signals common to both, which will indicate the structure of the dopamine polymer chain. Thus, in the spectrum corresponding to DOPA = βETA, signals are observed in the aromatic zone, between 6.5 ppm and 7 ppm, which can only correspond to the protons of the indole formed in the polymerization process (blue square symbol). However, as mentioned above, this is not the only structure that makes up the polymer, and two intense signals are observed in both spectra at 3.5 ppm (blue star symbol) and a third at 2.0 ppm (blue triangular symbol), which are aliphatic and would correspond to the protons of the other two structures proposed for this polymer. From one of these same monomers, a broad signal at 5 ppm is observed in both spectra, corresponding to the NH of pyrrolidine (blue circle symbol). Both β-cyclocitral and cinnamaldehyde have aldehyde groups in their structures, and these essential oils will be incorporated into the polydopamine structure through a reaction between these electrophilic groups and the nucleophilic groups of the polymer, which are OH, to form a cyclic acetal, or through the amine to form an imine, as depicted in Fig. 3 a. Because of the complexity of the NMR spectrum and the lower concentration of the oil compared to the polymer, it is difficult to clearly assign these new functional groups that have formed. Specifically, the proton of an acetal should appear near 6.5 ppm, but this coincides with the aromatic zone, making it difficult to distinguish it from those already mentioned. Similarly, in the case of DOPA = CIN, a proton at 9.5 ppm can be observed, which could be assigned to the imine, but the protons of the OH or NH of the indole could also be seen in this zone. In any case, the incorporation of essential oils is clear, as the characteristic signals for each of them can be observed. For example, in DOPA = CIN, an increase in the most unshielded signals is observed in the aromatic zone, among which a doublet characteristic of the aromatic AB system stands out due to coupling at 7.7 ppm (red pentagonal symbol) and a singlet at 7.4 attributable to the double bond (red hexagonal symbol)., while in DOPA = βETA, signals at 0.9 (green pentagonal symbol) and 1.4 (green hexagonal symbol) are clearly associated with the methyl groups, CH_3_, of the β-cyclocitral structure. This undoubtedly demonstrates the incorporation of essential oils into the polymer structure. We find something very similar in the FTIR spectra (Fig. 3c). Both essential oils show the most characteristic signals corresponding to the aldehyde group, such as the C=O stretching frequencies at 1671 cm^−1^ and the two signals corresponding to the stretching vibrational modes of the aldehyde proton around 2850 cm^−1^ and 2750 cm^−1^, in both cases. These three signals disappear in both DOPA = CIN and DOPA = βETA, indicating the disappearance of the aldehyde group. On the contrary, in DOPA = βETA and DOPA = CIN, a signal appears at 1630 cm^−1^ and 1618 cm^−1^, respectively, which can be attributed to the formation of the imine, indicating that this may be the form of binding to the polymer. Acetal groups have as a characteristic signal an intense stretching band due to the vibration of the C-O bonds at 1200 cm^−1^, and the intensity of the broad band between 3500 cm^−1^ and 2500 cm^−1^, associated with the stretching frequency of the OH groups of the dopamine ring, should disappear or decrease. The 1200 cm^−1^ signal does not appear to be observed in the spectra, nor does the OH signal disappear, so everything seems to indicate that the bond is through an imine bond. Therefore, although the structure of the dopamine polymer functionalized with essential oils is very complex, the combination of NMR and FTIR indicates that these have been incorporated into the structure and that this is most likely through an imine-type bond.

To evaluate GER incorporation into the nanoparticles, the encapsulation efficiency (EE%) and loading capacity (LC%) were determined using the equations described in Section 2.6. Both formulations demonstrated successful GER encapsulation, with values of 32 ± 2 % EE and 56 ± 2 % LC for dMSNs–GER, and 32 ± 2 % EE and 55 ± 1 % LC after coating with the functional polymer layer. These results indicate minimal GER loss during the coating process and confirm the robustness of the encapsulation method.

For comparison, a recent study on the encapsulation of thyme essential oil in dMSNs reported only ∼15 % encapsulation, despite using nanoparticles with a much higher surface area (394.8 m^2^ g^−1^) [30]. This contrast highlights that surface area alone does not determine loading capacity; hydrophobic interactions, pore architecture, and chemical bonding play essential roles. Similar trends have been observed in the encapsulation of lemongrass and clove oils in MSNs, where LC values of 33.75 % and 26.91 % were achieved despite a significantly higher surface area (1603.6 m^2^ g^−1^) [31]. [31].

Fig. 1a shows the Uv-vis spectra collected with different functionalized nanoparticles in aqueous suspension in the measurements from 190 nm to 400 nm. Both bio-functionalized DOPA coated nanoparticles exhibit spectra with the characteristic's peaks of each biomolecule confirming these attachments. In the case of dMSNs- DOPA = CIN the collected spectra showed the maximum abs at 290 nm with two minority peaks at 210 nm and 220 nm [32,33] and obtained a grafting efficiency of 41.0 ± 0.0 %. On the other hand, dMSNs-DOPA = βETA also showed the characteristic peak attributed to βeta-cyclocitral grafting exhibiting a broad peak with an absorbance maximum at 255 nm with grafting efficiency of 97.0 ± 0.0 %. Meanwhile, ungrafted dMSNs-DOPA did not show a significant uv-vis peak with only a slight increase in absorbance at 200 nm, as shown in other work with polydopamine coating materials [33].

### 3.2 Thermal properties

**Fig. 4:**
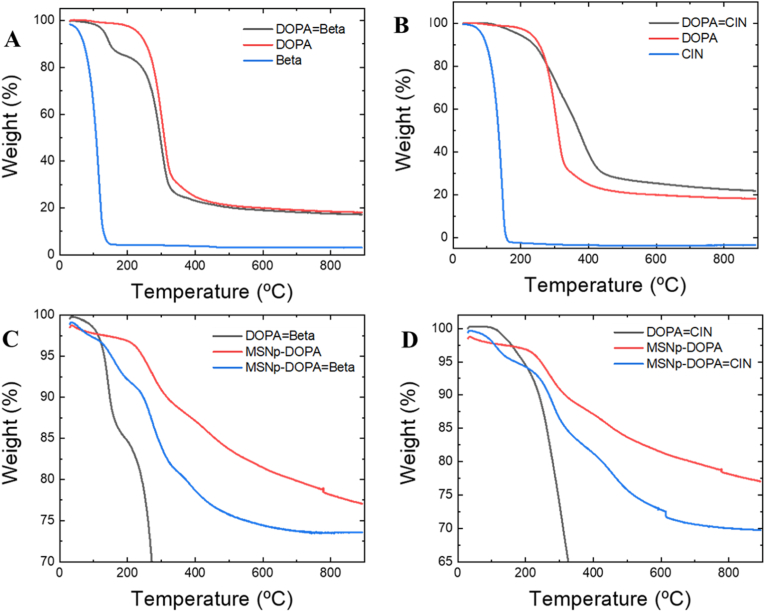
TGAgrams of A) β-cyclocitral (βETA), polydopamine (DOPA), polydopamine grafted with β-cyclocitral (DOPA = βETA). B) Cinnamaldehyde (CIN), polydopamine (DOPA), polydopamine grafted with cinnamaldehyde (DOPA = CIN). C) polydopamine grafted with β-cyclocitral (DOPA = βETA), Silica mesoporous nanoparticles coated with polydopamine (MSNp-DOPA), Silica mesoporous nanoparticles coated with polydopamine grafted with β-cyclocitral (MSNp-DOPA = βETA). D) polydopamine grafted with cinnamaldehyde (DOPA = CIN), Silica mesoporous nanoparticles coated with polydopamine (MSNp-DOPA), Silica mesoporous nanoparticles coated with polydopamine grafted with cinnamaldehyde (MSNp-DOPA = CIN).

Thermogravimetric Analysis (TGA) was used to evaluate the thermal stability and decomposition temperatures of the prepared materials and thermograms are shown in Fig. 4. The TGAgrams show that essential oils are highly volatile, evaporating almost immediately when the system begins to heat up (Fig. 4 A and 4 B). βETA is slightly more volatile than cinnamaldehyde. On the other hand, the dopamine polymer is perfectly stable up to 160°C–180 °C and has low water absorption, less than 1 %. The functionalization of polydopamine with the two essential oils shows that the loss of volatile oils shifts to higher temperatures, not before 100 °C, which is justified because the oils are covalently bound to the polymer and therefore hydrolysis is necessary to release and volatilize them. This has been previously observed by us when cinnamaldehyde was chemically bound to chitosan polymer. (referencia ultimo trabajo) As β-cyclocitral is more volatile, it is easy to determine from the TGAgram the amount of essential oil bound to the polymer, which in this case is 15 % of the total mass. In the case of cinnamaldehyde, this is not possible because, being less volatile, its evaporation begins to coincide with the thermal decomposition of the polydopamine. When polydopamine was bound to mesoporous silica nanoparticles (Fig. 4C and 4 D), it was observed that polymer degradation was delayed until temperatures reached 190 °C. Considering that at 900 °C only inorganic residues of the silica nanoparticles remain, we can estimate that the polydopamine load is 23 % of the total weight. The TGAgrams of the silica nanoparticles loaded with polydopamine functionalized with essential oils show the loss of oils at temperatures close to 100 °C, which indicates that they are covalently bonded to the polymer and ultimately leads to a greater loss of organic matter mass, as this corresponds to the mass of the polymer and the essential oil. Assuming that the amount of polymer mass is the same in MSNp-DOPA, by difference we can estimate the amount of essential oil contained in each of the nanoparticles, specifically 7 % in the case of MSNp-DOPA = CIN and 3.5 % in the case of MSNp-DOPA = βETA of the total mass. In the latter case, if we take into account the ratio between the polymer and the essential oil of β-cyclocitral, we see that it is 15.2 % with respect to polydopamine, which is in agreement with the amount estimated in the TGA of Fig. 4A calculated above. The differential scanning calorimetry experiments show no significant variation in any of the samples, indicating that there are no changes in the crystallinity of the polymer prior to its thermal degradation.

### 3.3 Surface area and pore size of dMSNs

N2 adsorption-desorption isotherms (Fig. 1B) were acquired to analyze the pore structure of the dMSNs, especially their mesoporosity and microporosity, in correlation with the TEM images [34]. The Brunauer-Emmett-Teller (BET) analysis of the nanoparticles indicated a specific surface area of 154 m^2^/g.

Various studies have reported differing surface area values for dMSNs, reflecting the sensitivity of their textural properties to synthesis conditions. For example, Zahiri et al. documented a specific surface area of approximately 150 m^2^/g for dendritic mesoporous silica nanoparticles synthesized under standard conditions [35]. In contrast, Wang et al. demonstrated that by varying the reaction time, while keeping reagent ratios and concentrations constant, it is possible to modulate the surface area significantly, obtaining values ranging from 200 to 488 m^2^/g [36].

The isotherm displayed a high relative pressure revealing the micro-mesoporous character of samples (type IV). The capillary condensation, accompanied by H1 hysteresis loops, indicated that the materials had ordered mesoporous structures [37]. The adsorption behavior of type IV isotherms in mesopores is determined by the adsorbent adsorptive interactions and the interactions among molecules in the condensed state. The sharp uptake observed in these isotherms at a relative pressure of approximately 0.8–1.0 is due to the nanoscale particle size and corresponds to interparticle adsorption or void volume between particles. As shown in other works with similar isotherm patterns, it is difficult to predict the shape of the mesopores because the hysteresis loops are not representative, but it is possible to confirm that the materials have micro-mesoporous morphology—long, flat adsorption with small hysteresis loops [34]. More information on pore size could be obtained from the peak observed in the Barrett-Joyner-Halenda (BJH) desorption plot of the pore size distribution (Fig. 1B), where a clear peak between 15 and 20 nm confirmed a pore radius size of 17.5 nm, which correlates well with the TEM images of the nanoparticles. Other works on dMSNs using IL imidazolium alkyls as structural steering agents with different chain lengths, showed a correlation of pore sizes from 3.5 to 21.4 nm, confirming that BIMIN used in our synthesis exerts the reported effect on pore size [38].

### 3.4 Dual and pH responsive release of bio-modified dopamine coated nanoparticles

The release profile of Geraniol (GER) confirms the pH-responsive behavior of the coated nanocarriers (Fig. 2B), showing a statistically significant increase (p < 0.0001) in GER release at pH 5 compared to pH 7. After 200 h, the cumulative GER release reached 94 % and 12 % for the coated systems at acidic and neutral pH, respectively. The release kinetics suggest that the mechanism is predominantly governed by the hydrolytic degradation of the dopamine-derived polymeric coating. This leads to an initial lag phase characterized by minimal GER diffusion, followed by a marked increase in release as the acidic environment triggers polymer disintegration. In contrast, under neutral pH conditions, a diffusion-controlled release mechanism predominates throughout the entire profile.

Uncoated nanoparticles did not exhibit significant differences in GER release between pH 5 and pH 7, further confirming the functional role of the DOPA-based coating. These findings align with previous studies, such as that by Jadidi et al., in which DOPA-coated hollow mesoporous nanoparticles demonstrated enhanced release of gefitinib under acidic conditions, achieving up to a 50 % increase in drug release due to pH-triggered polymer degradation [39].

Fig. 2C illustrates the dual release behavior of the novel DOPA-functionalized nanoparticles, confirming the cumulative release of both CIN and βETA. In both systems, a statistically significant (p < 0.05) enhancement in biomolecule release was observed under acidic conditions (pH 5) compared to neutral pH (pH 7), consistent with a pH-responsive release mechanism.

For CIN, the release profile displayed an initial burst phase, with approximately 30 % of the total content released within the first 6 h. This was followed by a sustained release phase, reaching 50 % cumulative release at pH 5 versus only 29 % at pH 7. The observed biphasic behavior is indicative of a mechanism primarily driven by the hydrolysis of imine bonds formed between the aldehyde moieties of CIN and the amine groups of polydopamine during polymerization. In contrast, βETA exhibited a triphasic release profile. The initial phase was governed by passive diffusion, followed by an accelerated release after 144 h, attributable to the gradual hydrolysis of terpene-polymer linkages under acidic condition [17]s. This led to a final release of up to 81 % at pH 5, compared to 48 % at pH 7.

The observed pH-responsive release of the encapsulated molecules, such as GER and grafted molecules (CIN and βETA), demonstrates that the integrity and degradation of the dopamine-derived polymeric coating are highly susceptible to environmental variations. Specifically, the sharp increase in cumulative release at pH 5 compared to pH 7 (94 % vs 12 % GER) highlights the critical role of soil pH in triggering the hydrolytic disintegration of the nanocarrier matrix and the hydrolysis of imine bonds [17]. In an agro-environmental context, this suggests that the acidic microenvironments often found in the soil rhizosphere or associated with microbial metabolic activity could significantly accelerate the release rate [40,41]. Furthermore, while not explicitly studied, the presence of microbial enzymes—particularly esterases, amidases, or those capable of phenol oxidation—found in the soil microbiome may further catalyze the degradation of the DOPA coating, altering the predominant release kinetics from a diffusion-controlled to an accelerated, enzyme-mediated mechanism, thereby affecting the final bioavailability and efficacy of the active compounds [42].

Four mathematical models were employed to analyze the kinetic data in order to get a deeper understanding of the terpenes and apocarotenoid release process from multifunctional nanomaterials. Analyzing the kinetics of biomolecules in PBS at pH7 and pH5 involved using Eqs. (5), (6). Table 1 presents the recordedr esults for all described biomolecule kinetic models. Based on the best fit with the R^2^ value, we observed that the most of them adhere to the Korsmeyer-Peppas models main while dMSNs-GER-DOPA-Modif pH 5 and βETA release from biofunctionalized nanoparticles displayed a zero-order release profile. The release mechanism of GER from unmodified nanoparticles was considered to be Fickian diffusion, as shown by the release exponent (n)values being smaller than 0.45. The leaching of GER molecules from the surface of the nanoparticle pores may have occurred, leading to the instantaneous release and subsequent diffusion of terpene from the multichannel. However, GER release followed a different release profile with the modified nanoparticles, in which the nanoparticles showed a non-Fickian release (n = 0.45–1) mechanism mainly due to erosion of the polymer layer, coinciding with the release profile obtained and subsequent analysis. A similar trend was observed in the case of βETA release, where the kinetics followed a non-Fickian profile due to the greater degradability of the apocarotenoid bond to polydopamine, confirmed by the release curve, where greater release at pH 5 is related to greater susceptibility to covalent bond breakage and therefore greater stimuli-responsive capacity.

**Table 1:** Kinetics analysis of GER, CIM and βETA release data from proposed nanomaterials using different kinetic models.

Nanomaterial	Zero-Order (R^2^)	First-Order (R^2^)	Higuchi model (R^2^)	Korsmeyer-Peppas (R^2^)	Korsmeyer-Peppas (n)	Release Mechanism
dMSNs-GER pH 5	0,382717	0,391910	0,510003	**0,909459**	0,030943	Fickian difussion
dMSNs-GER pH 7	0,545450	0,571277	0,717365	**0,884458**	0,178987	Fickian difussion
dMSNs-GER-DOPA-Modif pH 5	**0,923172**	0,884648	0,820040	0,899728	0,839068	Anomalous transport
dMSNs-GER-DOPA-Modif pH 7	0,908379	0,902642	0,802761	**0,917842**	1,076002	No Fickian transport (Class II)
CIN pH 5	0,878727	0,932803	0,967044	**0,981380**	0,248372	Fickian difussion
CIN pH 7	0,819561	0,843150	0,937567	**0,975230**	0,241315	Fickian difussion
βETA pH 5	0,909083	0,827176	0,803857	**0,915316**	1,049146	No Fickian transport (Class II)
βETA pH 7	**0,907555**	0,890034	0,801880	0,898610	0,936212	Anomalous transport

In summary, the newly developed dMSNs exhibit a dual-release capability with clear stimulus-responsive behavior, enabling differentiated release kinetics that may enhance antifungal efficacy. Two distinct formulations were characterized: dMSNs-GER-DOPA = CIN, which achieved a cumulative release of 94 % of Geraniol (GER) and 50 % of Cinnamaldehyde (CIN) within 200 h; and dMSNs-GER-DOPA = βETA, which released 94 % of GER and up to 81 % of (βETA over the same period. These release profiles highlight the potential of this nanoplatform as a tunable delivery system for combinatorial antifungal strategies.

### 3.5 Broad spectrum and high antifungal activity of nanoparticles coated with biomodified dopamine

**Fig. 5:**
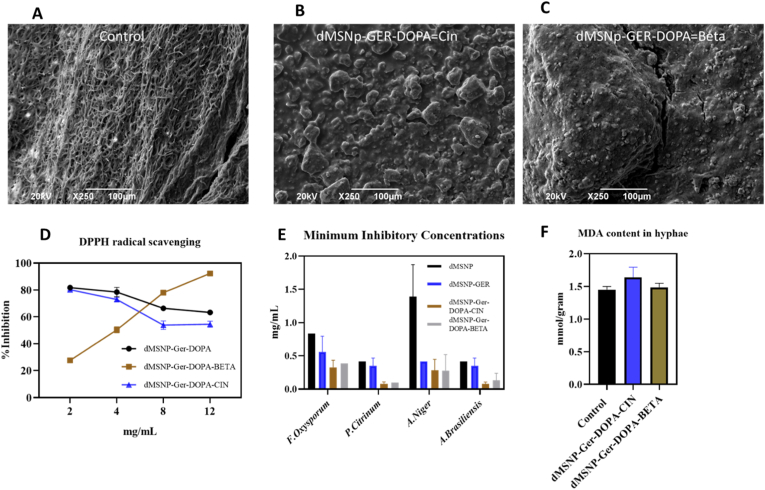
A-C) SEM images of untreated fungi hyphae and treated with novel nanodecives, D) antioxidant activity of nanoparticles with DPPH radical scavenging, E) Antifungal evaluation of nanoparticles Treatments were performed in liquid PDB medium multiwell plates at range of doses from 5 mg/mL up to 0.05 mg/mL. F) MDA content of treated hyphae. Data was generated as a result of three independent experiments.

Fig. 5 illustrates the antifungal activity, related to 100 % of fungal inhibition, of the synthesized nanomaterials against a panel of phytopathogenic fungi across various culture conditions. A consistent trend was observed among all fungal strains, whereby dopamine-coated, biomodified nanoparticles exhibited significantly enhanced antifungal effects, confirming the effectiveness of the functionalization strategy.

dMSNs demonstrated baseline antifungal activity across all tested species, with particularly notable effects against *Penicillium citrinum* and *Aspergillus brasiliensis*, each exhibiting a minimum inhibitory concentration (MIC) of 0.417 mg/mL. In contrast, reduced activity was observed against *Aspergillus niger*, with an MIC of 1.389 mg/mL. This intrinsic antifungal activity may be attributed to the presence of silanol and hydroxyl groups on the nanoparticle surface. These groups are known to interact with cell wall components, such as lipopolysaccharides, via hydrogen bonding, leading to disruption of membrane integrity and subsequent cell lysis [43,44]. Additionally, electrostatic interactions between the positively charged dMSNs and the negatively charged fungal membranes may contribute to membrane destabilization, disruption of transmembrane energy cycles, or even oxidative damage. Prior studies have also implicated the interaction of nanoparticle surfaces with thiol (-SH) groups on membrane proteins as a lytic mechanism [45].

A statistically significant enhancement in antifungal activity (p < 0.001) was observed following encapsulation of Geraniol (GER), especially notable in *A. niger*, where MIC values dropped from 1.389 mg/mL (empty dMSNs) to 0.417 mg/mL (dMSNs-GER). Interestingly, GER alone did not exhibit antifungal activity at the tested concentrations (≤2 mg/mL), suggesting a synergistic effect resulting from its nanoencapsulation. Across all fungi, GER-loaded dMSNs showed improved antifungal performance, with *P. citrinum* exhibiting the highest sensitivity (p = 0.309).

Coating GER-loaded dMSNs with biofunctionalized dopamine polymers (DOPA = CIN or DOPA = βETA) further enhanced antifungal efficacy, as reflected in significantly lower MIC values. Specifically, *P. citrinum* and *A. brasiliensis* were the most susceptible strains, with MICs of 0.081 and 0.078 mg/mL, respectively, for the dMSNs-GER-DOPA = CIN formulation, and 0.096 and 0.131 mg/mL, respectively, for dMSNs-GER-DOPA = βETA. Although not all fungal strains showed statistically significant differences between new nanoparticles (p = 0.9708), a clear downward trend in MIC values was observed across the board, showing the broad-spectrum potential of these dual-functional nanoparticles.

The synergistic co-delivery of GER with CIN or βETA within a single nanocarrier appears to potentiate antifungal activity displaying significative differences respect unmodified nanoparticles with slight higher differences in the case of CIN modified (p = 0.0104) respect βETA modified (p = 0.0309), confirming the success of this dual-release nanoplatform as a promising strategy for biocontrol of pathogenic fungi.

Furthermore, the enhanced antifungal activity of the novel nanoparticles was corroborated by scanning electron microscopy (SEM), as shown in Fig. 5 A. Untreated *Fusarium oxysporum* mycelia exhibited well-preserved morphological features, including filamentous, thread-like hyphae with clearly defined porosity and branching structures. In contrast, SEM images of *F. oxysporum* treated with both nanoparticle formulations revealed extensive structural disruption. The polysaccharide, and the protein matrix of the mycelium appeared completely degraded, with the formation of amorphous aggregates and loss of hyphal integrity, indicating a breakdown of cellular architecture, supporting the strong antifungal effect of the treatments. These morphological alterations are consistent with previously reported effects observed in fungi exposed to other metallic nanomaterials, such as copper and silver, which have also demonstrated cell wall and mycelial degradation upon nanoparticle exposure. The SEM-based evidence thus reinforces the proposed mechanism of action of the biofunctionalized dMSNs, confirming their capacity to induce fungal cell collapse through direct structural disruption [46,47].

### 3.6 Different antioxidant behavior of multifunctional nanoparticles

Antioxidants can be used in seed dressing to improve seed quality, longevity, and stress tolerance during storage and early growth. Common antioxidant compounds include superoxide dismutase, catalase, glutathione peroxidase, ascorbic acid, tocopherol, glutathione, flavonoids, polyphenols, and carotenoids. These compounds help protect seeds from oxidative stress, which can damage them during storage and germination [48].

To validate the antioxidant properties of the synthesized nanoparticles and further support their potential as multifunctional antifungal systems with antioxidant capacity, we conducted a DPPH radical scavenging assay. The results, presented in Fig. 5 D-demonstrate concentration-dependent antioxidant behavior across the tested formulations. Nanoparticles functionalized with β-cyclocitral exhibited a dose-dependent increase in antioxidant activity, reaching a maximum inhibition of 92 % at 16 mg/mL. In contrast, nanoparticles modified with cinnamaldehyde displayed an inverse trend: their highest antioxidant capacity (54 %) was observed at a lower concentration (2 mg/mL), with subsequent decreases at higher concentrations. This behavior closely mirrors that of uncoated dMSNs, suggesting a saturation or inhibitory threshold.

Interestingly, the dMSNs-DOPA = CIN formulation exhibited a marked decline in antioxidant activity with increasing nanoparticle concentration. This counterintuitive behavior could be explained by the “antioxidant paradox,” whereby high concentrations of cinnamaldehyde may promote the formation of peroxides or reactive intermediates, thereby increasing the oxidative potential of the medium rather than reducing it [49,50]. Nevertheless, both biofunctionalized nanoparticles achieved comparable antioxidant activity at intermediate concentrations (5 mg/mL), with DPPH inhibition values of approximately 73 %. These findings are in agreement with previous studies. For example, Dong et al. reported similar antioxidant activity (∼80 % inhibition at 5 mg/mL) using silica nanoparticles encapsulating gallic acid in edible films. Likewise, Hosseini and co-workers demonstrated that cinnamaldehyde–chitosan nanoparticles embedded in bifunctional films displayed modest DPPH inhibition (∼16 %) at equivalent nanoparticle concentrations, further supporting the antioxidant potential of terpene-based nanomaterials [51].

#### 3.6.1 Antioxidant properties of nanoparticles in wheat seeds

**Fig. 6:**
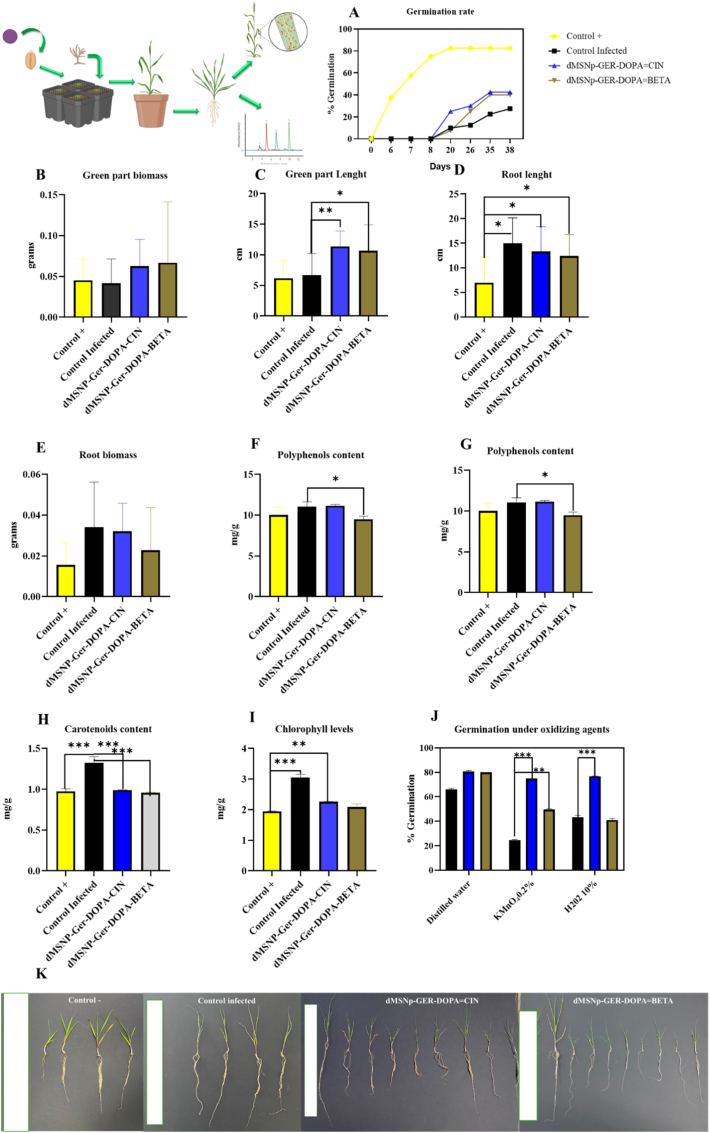
A) germination/seedling emergence. B-I) Physiological and response paramenters of treated plants; grams (grams of plant part), mg/g (mg of correspond plant metabolite/gram of dried plant), cm (centimeters of plant part) (k) Images of treated plants after 38 days of sowing. Data was generated as a result of three independent experiments. The result showed has been statistically evaluated using two-way ANOVA with post hoc analysis (∗- p < 0.05, ∗∗-p < 0.01 and ∗∗∗-p < 0.001).

Both H_2_O_2_ (hydrogen peroxide) and KMnO_4_ (potassium permanganate) are two strong oxidizing agents that can inhibit seed germination by damaging critical cellular processes. KMnO_4_, being a strong oxidant, exerts its toxicity mainly by disrupting the electron transport chain in the embryo's mitochondrial respiration, analogous to its effect on Photosystem II, which stops the synthesis of ATP crucial for radicle growth. In addition, its high oxidative capacity inactivates enzymes essential for germination (such as amylases and lipases) by reacting with sulfhydryl (-SH) groups in their active sites, preventing the mobilization of energy reserves (starches, lipids), and consequently blocking development [52,53]. Although H_2_O_2_ can act as a signaling molecule at low doses, at high concentrations it also becomes an oxidative stress agent that damages membranes, lipids, and proteins (including enzymes) through reactive oxygen species, thus compromising cell viability and the seed's ability to complete the germination process [54]. The results showed in Fig. 6j clearly demonstrate the toxic effect of oxidizing agents and the protective efficacy of nanoparticles. Germination is drastically reduced by exposure to 0.2 % KMnO_4_ (falling from approximately 65 % to approximately 25 %) and, to a lesser extent, by 10 % H_2_O_2_ (falling to approximately 43 %). However, the dMSNs-DOPA = CIN (blue bar) and dMSNs-DOPA = βETA (brown bar) nanoparticles exert a powerful and antioxidant effect significantly reversing the inhibition: the dMSNs-GER-DOPA = CIN nanoparticle was the most effective (p < 0.0001), restoring the germination rate to levels close to 80 % in both oxidizing treatments (exceeding control levels), while dMSNs-DOPA = βETA also offered protection, although with less efficacy, especially under KMnO4. In summary, the study validates the ability of these nanoparticles to counteract oxidative stress induced by KMnO_4_ and H_2_O_2_, promoting seed viability and growth (see Fig. 6).

**Fig. 7:**
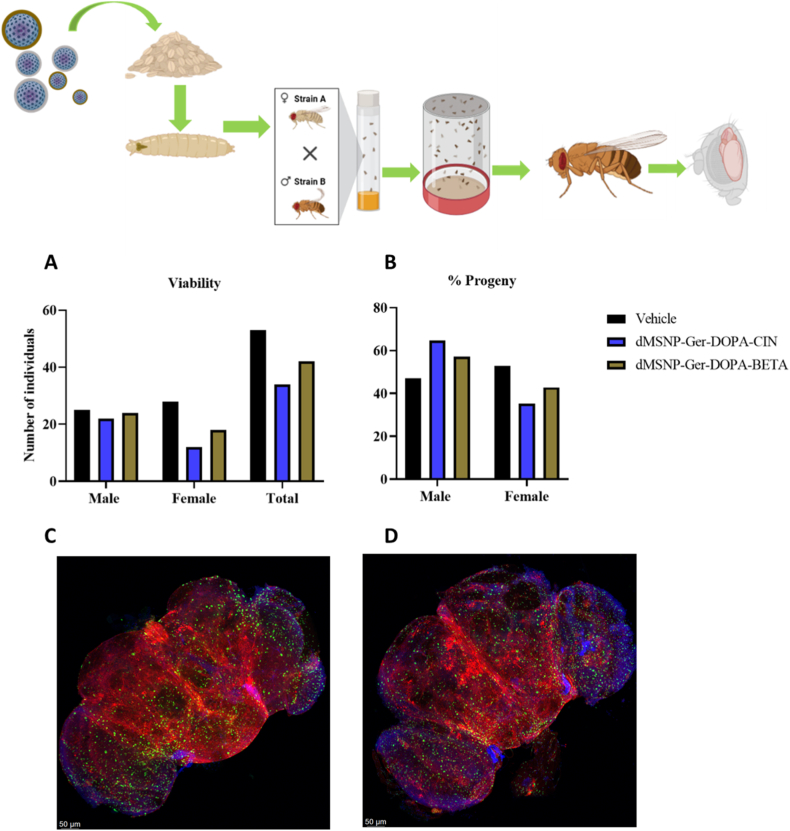
Biosafety profile of novel nanoparticles in *Drosophila Melanogaster* A) Viability of treated individuals. B) Percent of new progeny after nanoparticles treatment. C) Female brain of treated *Drosophila Melanogaster.* D) Male brain of treated *Drosophila Melanogaster*.

### 3.7 Successful antifungal activity of nanoparticles in wheat seeds

Seed germination rate is one of the important indicators in measuring seed quality and seed germination ability, and it is also an important basis for evaluating the growth potential and planting effect of seeds [55]. Furthermore, to evaluate the activity and efficacy of an antifungal seed treatment, the substance or treatment being evaluated must increase the number of plants that germinate in the presence of the phytopathogenic fungus [56].

Fig. 5 A showed how the germination of wheat seeds varied significantly among treatments, highlighting the impact of *Fusarium graminearum* infection and the protective potential of functionalized nanoparticles. The results were statistically evaluated using two-way multicomparison ANOVA with post hoc analysis (∗- p < 0.05, ∗∗-p < 0.01 and ∗∗∗-p < 0.001). Untreated, uninoculated seeds (Control) exhibited the highest germination rate starting the germination from 6 days with 35 % of germination achieving a germination rate of 82.5 % in day 20 confirming the viability and the successful physiological state of the wheat seeds. On the other hand, seeds inoculated with untreated *F. graminearum* (infected control) showed a very significative decrease (p < 0.0001), confirming the detrimental effect of the pathogen on seed viability, delaying germination until day 20 and decreasing the germination rate to 27.5 %, with a decrease in germination percentage of 55 %, confirming the strong presence of pathogenic fungi in the sowing. Different works reported germination reduction of wheat infested seeds with *F. graminearum* with values oscillating between 25 % up to 80 % depending the amount of inoculum and conditions [57].

Successful antifungal activity in seed dressing nano-treatment was confirmed with germination rates values observed in Fig. 5A. Treatment with cinnamaldehyde-functionalized nanoparticles (dMSNs-GER-DOPA = CIN) significantly improved germination in comparison to the infected control (p = 0.0005) achieving maximum germination rate of 42 % with an increase of 15 % respect untreated and infested seeds, indicating a protective aforementioned antifungal effect likely due to cinnamaldehyde's ability to disrupt fungal cell membranes and inhibit mycotoxin synthesis [58].

Despite the strong delay in germination typically induced by fungal infection, seeds treated with the biofunctionalized nanoparticles initiated germination concurrently with the untreated control group. By day 20, the dMSNs-GER-DOPA = CIN formulation had already produced a 2.5 % increase in germination, which progressively rose to 42.5 % by day 38. A similar trend was observed for the β-cyclocitral-functionalized formulation (dMSNs-GER-DOPA = βETA), which achieved a statistically significant improvement in germination rate compared to the infected control (p = 0.016), reaching a maximum of 40 %, representing a 12.5 % increase.

Although the absolute germination values obtained with the CIN-functionalized system were slightly higher, no statistically significant differences were observed between both nanoformulations regarding seed protection. While the direct antifungal mechanism of β-cyclocitral remains poorly characterized, its known role as a plant-derived apocarotenoid involved in stress signaling suggests an indirect mechanism—potentially via modulation of plant defense pathways and enhancement of seedling vigor, particularly root establishment [59].

In addition to the aforementioned antifungal effect, which results in increased germination promotion, other molecular mechanisms could be involved synergistically through the use of this new nanoparticles. Different mechanisms may explain the pro-germinative effect of the proposed treatments. Silica nanoparticles may improve seed germination and seedling growth by modulating antioxidant enzymes and mitigating lipid peroxidation [60]. Recently, many studies have shown how terpenes such as CIN and GER, at lower doses, could increase seed germination by triggering biochemical changes, such as increased production of phytohormones (gibberellic acid (GA3)), and activating stress-related genes that improve the ability of seeds to repair themselves, enhancing the synthesis of important proteins and antioxidants, stimulating metabolic activity, and influencing cell wall structure, ultimately leading to increased germination potential [61]. Moreover, Dickinson and co-workers determined that β-cyclocitral is a broadly effective root growth promoter in both monocots and eudicots and could be a valuable tool to enhance crop vigor under environmental stress [59].

Despite the biochemical, hormonal, and molecular differences in the mechanisms of action of cinnamaldehyde and β-cyclocitral, both treatments were equally effective in mitigating the deleterious effects of fungal infection on germination.

### 3.8 Phytosecurity profile of nanoparticles

Before confirming the use of new nano-treatments as a phytosanitary product, despite their antifungal activity, it is mandatory to demonstrate whether the proposed treatment has a satisfactory phytosafety profile without altering its physiological and morphological parameters. In order to evaluate it we have measure different morphological and physiological parameters to get conclusion about the activity in plant metabolism [11]. Fig. 6 J showed the images of plant collected samples and the graph of their related measures.

These findings suggest that *Fusarium oxysporum* infection, despite inducing mortality in a subset of plants, may not significantly impair above-ground biomass accumulation in surviving individuals during the initial 38 days post-inoculation. This could be attributed either to the selection of inherently more resilient seedlings or to early compensatory growth responses. Notably, treatments with biofunctionalized nanoparticles, particularly dMSNs-GER-DOPA = CIN, appear to promote vegetative growth, possibly by alleviating the sublethal stress induced by the pathogen. This observation aligns with previous reports suggesting that certain nanomaterials can enhance plant growth under biotic stress by facilitating nutrient uptake or modulating stress-response signaling pathways [62].

In parallel, shoot length measurements revealed no significant differences between the uninfected control (6.2 cm) and the infected control group (6.7 cm), suggesting that *F. oxysporum* at the tested inoculum level (3000 spores/seed) does not markedly suppress early shoot elongation in surviving plants. However, both dMSNs-GER-DOPA = CIN and dMSNs-GER-DOPA = βETA treatments elicited a statistically significant increase in shoot length compared to the infected control (p < 0.05 and p < 0.01, respectively), indicating a potential growth-promoting effect of the nanoparticles under pathogen stress. The absence of significant differences between control and nanoparticle-treated groups may reflect either a lack of impact on this particular trait or the need for a longer evaluation period to observe more pronounced effects.

Root length, by contrast, showed a clear and statistically significant reduction (p < 0.05) in the infected control group (6.5 cm) compared to the uninfected control (15 cm), consistent with the well-documented root-damaging effects of *Fusarium* infection, which impairs water and nutrient uptake (e.g., Wang et al., 2019) [63]. Importantly, both nanoparticle treatments restored root growth to levels comparable to or even exceeding that of the healthy control group.

Root biomass data closely paralleled the patterns observed for root length. Infected control plants exhibited a reduction in root biomass relative to the uninfected control, although this difference was not statistically verified within the dataset. It is plausible that the surviving plants partially compensated for pathogen stress through activation of systemic defense responses, which may lead to root system reinforcement aimed at restoring stability and improving nutrient and water uptake efficiency. In contrast, both dMSNs-GER-DOPA = CIN and dMSNs-GER-DOPA = βETA treatments demonstrated a consistent trend toward increased root biomass, in some cases approaching or surpassing the levels observed in the uninfected control group. These findings suggest that nanoparticle treatment supports the development of a more robust root system, which is critical for overall plant vigor, resilience under biotic stress, and improved tolerance to soil-borne pathogens. The observed enhancement in root biomass aligns with previous reports on nanomaterial-mediated stimulation of root growth, commonly attributed to improved micronutrient availability, localized modulation of hormonal pathways (e.g., auxin signaling), or direct stimulation of root meristematic activity [64].

Polyphenol content, a well-established biochemical marker of plant defense activation [65], was evaluated to assess the systemic response to fungal infection and nanoparticle treatment. As shown in Fig. 6 G, polyphenol levels were slightly elevated in the infected control group (11.4 mg/g) compared to the uninfected control (10.9 mg/g), although the difference was not statistically significant. This moderate increase is consistent with typical plant responses to pathogen challenge, wherein polyphenols function as antioxidants and antimicrobial agents [66].

Interestingly, both nanoparticle treatments exhibited reduced polyphenol levels compared to the infected control. The dMSNs-GER-DOPA = CIN group showed 11.3 mg/g, whereas the β-cyclocitral-functionalized formulation (dMSNs-GER-DOPA = βETA) exhibited a significant decrease (9.9 mg/g; p < 0.05). These results suggest that although the nanoparticles are effective in suppressing infection, they do not elicit a broad upregulation of polyphenol biosynthesis. Alternatively, it is possible that other defense pathways, rather than general polyphenolic responses, are preferentially activated under nanoparticle-mediated protection [67].

Carotenoids, multifunctional metabolites involved in photosynthesis, photoprotection, and signaling, also play a protective role under stress conditions by scavenging reactive oxygen species and quenching singlet oxygen [68]. The results indicate a marked increase in carotenoid content in infected but untreated seeds, supporting previous findings in *Alternaria alternata*-infected plants, where exposure to pathogen volatiles enhanced photosynthetic efficiency and chloroplast function [69]. By contrast, carotenoid levels in nanoparticle-treated seeds remained comparable to those of uninfected controls. This suggests that the nanoparticle-induced defense mechanisms may not rely on carotenoid biosynthesis or that sufficient basal carotenoid levels are maintained for functional photoprotection, even under biotic stress.

Chlorophyll content, another key indicator of plant physiological status, plays an essential role in light absorption, growth regulation, and defense signaling [70]. Notably, *F. oxysporum*-infected seeds exhibited a significant increase in total chlorophyll content (3.2 mg/g; p < 0.001) relative to uninfected controls (1.9 mg/g), potentially as a compensatory response to stress-induced metabolic demand or pathogen-derived elicitors such as fusaric acid [71]. Treatment with biofunctionalized nanoparticles effectively mitigated this chlorophyll overaccumulation. The dMSNs-GER-DOPA = CIN and dMSNs-GER-DOPA = βETA groups showed values of 2.0 mg/g and 2.3 mg/g, respectively—both significantly different from the infected control, with the CIN-functionalized formulation showing a highly significant normalization (p < 0.01).These findings show the capacity of the designed nanocarriers to preserve photosynthetic homeostasis in infected seedlings, likely by controlling pathogen proliferation and maintaining physiological integrity. This aligns with growing evidence that nanomaterials can stabilize chlorophyll levels and improve photosynthetic performance under abiotic and biotic stress conditions [72].

### 3.9 Biosafety evaluation of nanocarriers in insect model Drosophila Melanogaster

Plant Protection Products (PPPs), commonly referred to as pesticides, represent one of the most effective tools for safeguarding crops against pests and pathogens. Their use is essential for improving crop management, protecting plant products, and enhancing agricultural productivity. According to EU regulatory frameworks, PPPs constitute a key strategy for preventing damage caused by harmful organisms, including weeds, and for securing yield stability and food safety [73].

To classify a formulation as a PPP, the active substance must be officially approved and all components of the final marketed product must demonstrate efficacy against the designated target organism without inducing adverse phytotoxic effects [74]. Prior to approval, the active compound must also meet stringent safety criteria, including demonstrated effectiveness against the target species and no unacceptable risk to human or animal health or to the environment. Furthermore, the substance must not induce negative impacts on non-target species, biodiversity, or ecosystem functions, and must not cause undue suffering in vertebrate test organisms [75].

To ensure environmental safety, a wide range of non-target organisms are employed in ecotoxicological evaluations of PPPs. Commonly used models include aquatic invertebrates such as *Daphnia magna*, *Chironomus* spp., *and Americamysis bahia*, as well as terrestrial organisms like earthworms, coleopterans, and spiders. These species are essential for assessing bioaccumulation potential, biotransformation rates, and chronic effects under environmentally relevant exposure conditions [76].

Recently, *Drosophila melanogaster* has emerged as a model organism for evaluating the ecotoxicological and endocrine-disrupting potential of novel PPPs. Its short life cycle, genetic tractability, and well-characterized biology make it particularly suitable for high-throughput toxicity screening. Notably, *D. melanogaster* has been successfully employed in studies assessing insecticidal activity and developmental toxicity, offering insights into both lethal and sublethal effects [77].

In the current study, the viability of *D. melanogaster* was assessed following exposure to two nanoparticle formulations: dMSNs-GER-DOPA = CIN and dMSNs-GER-DOPA = βETA. The data revealed in Fig. 7. no significant differences in total adult viability, sex distribution, or emergence rates when compared to vehicle controls, indicating that these formulations do not induce overt toxicity under the tested conditions. A slight but consistent decrease in female numbers was observed across nanoparticle treatments, potentially suggesting subtle sex-specific effects on development or survival. However, this deviation did not reach statistical significance and does not reflect generalized toxicity.

In contrast, previous studies on the insecticide Oberon demonstrated up to 80 % mortality in *D. melanogaster*, underscoring the importance of comparative bioassays in establishing the relative safety of novel PPP candidates. Collectively, our data indicate a favorable in vivo biosafety profile in *D. melanogaster* and support further development as plant protection candidates and hold promise for further development as safe and effective plant protection agents [78].

## 4 Conclusions

This study presents the synthesis and characterization of two innovative multifunctional nano-delivery systems, dMSNs–GER–DOPA = CIN and dMSNs–GER–DOPA = βETA, engineered on a dMSNs platform. These constructs are designed to synergistically combine antifungal efficacy with plant-growth promotion by encapsulating GER and covalently integrating CIN or βETA via a polydopamine surface coating. The resulting systems exhibited a distinct pH-responsive, dual-release mechanism, achieving a robust 94\% cumulative GER release at pH 5, alongside the controlled release of the grafted compounds (up to 50 % CIN and 81 % for βETA). Mechanistically, these nanoparticles demonstrated broad-spectrum antifungal activity against major phytopathogens, with minimum inhibitory concentrations (MICs) as low as 0.07 mg\mL, and successfully induced significant mycelial disruption. Beyond their antimicrobial role, they promoted seed germination and early plant development in pathogen-infected models, evidenced by the restoration of physiological markers and notable antioxidant activity up to 92 % DPPH inhibition for the βETA -functionalized nanoparticles. Crucially, biosafety was established in vivo using the *Drosophila melanogaster* model, showing no significant toxicity. These comprehensive findings position these novel nanoagro-formulations as highly promising candidates for developing next-generation antifungal agents, subject to future validation regarding their long-term stability, persistence, greenhouse/field efficacy, and economic scalability. For this reason, the future works must focus on mandatory greenhouse and field-scale validation to confirm long-term safety and efficac under real-world conditions. Simultaneously, the research must address the cost-effectiveness and manufacturing scalability of nanoparticles. These steps are essential to transition the promising lab-based technology into a viable, accessible agricultural solution.

## CRediT authorship contribution statement

**Maria Paz García-Simarro:** Methodology. **Maria Mondéjar-López:** Methodology. **Joaquin C. García-Martínez:** Methodology. **Antonio Cuesta-Casas:** Methodology. **Sergio Casas-Tintó:** Methodology. **Oussama Ahrazem:** Writing – review & editing, Visualization. **Lourdes Gómez-Gómez:** Project administration. **Enrique Niza:** Writing – review & editing, Writing – original draft, Validation, Supervision, Project administration, Methodology, Investigation, Funding acquisition, Formal analysis, Data curation, Conceptualization.

## Declaration of competing interest

The authors declare that they have no known competing financial interests or personal relationships that could have appeared to influence the work reported in this paper.

## Data Availability

Data will be made available on request.

## References

[1] Li Y. (2024). Metal-organic frameworks for sustainable crop disease management: current applications, mechanistic insights, and future challenges. J. Agric. Food Chem..

[2] Luo X. (2023). Nanomaterial size and surface modification mediate disease resistance activation in cucumber (Cucumis sativus). ACS Nano.

[3] Wang J. (2024). Lignin/surfactin coacervate as an eco-friendly pesticide carrier and antifungal agent against phytopathogen. ACS Nano.

[4] Chen X. (2024). Selenium nanomaterials enhance sheath blight resistance and nutritional quality of rice: mechanisms of action and human health benefit. ACS Nano.

[5] Sandri S., Hussein H., Alshyab N., Sagatowski J. (2023). The European green deal: challenges and opportunities for the Southern mediterranean. Mediterr. Polit..

[6] Mondéjar-López M., García-Simarro M.P., Navarro-Simarro P., Gómez-Gómez L., Ahrazem O., Niza E. (2024). A review on the encapsulation of ‘eco-friendly’ compounds in natural polymer-based nanoparticles as next generation nano-agrochemicals for sustainable agriculture and crop management. Int. J. Biol. Macromol..

[7] Malekmohammadi S. (2022). Nonordered dendritic mesoporous silica nanoparticles as promising platforms for advanced methods of diagnosis and therapies. Mater. Today Chem..

[8] Li X., Yang S., Luan Y., Wang D., Du X. (2024). Polydopamine modification on dendritic porous silica surface for efficient adhesion of functional nanoparticles. Colloids Surfaces A Physicochem. Eng. Asp..

[9] Mondejar M., Niza E. (2024). New gel from a water-soluble carboxymethyl chitosan-Cinnamaldehyde Schiff base Schiff base derivative as an effective preservative against soft rot in ginger.

[10] Modéjar-lópez M. (2022). Chitosan nanoparticles loaded with garlic essential oil : a new alternative to tebuconazole as seed dressing agent. Carbohydr. Polym..

[11] Mondéjar-López M., López-Jimenez A.J., Ahrazem O., Gómez-Gómez L., Niza E. (July 2022). Chitosan coated - biogenic silver nanoparticles from wheat residues as green antifungal and nanoprimig in wheat seeds. Int. J. Biol. Macromol..

[12] Wang S. (2017). Do quaternary ammonium monomers induce drug resistance in cariogenic, endodontic and periodontal bacterial species?. Dent. Mater..

[13] Mondejar M. (2022). Chitosan nanoparticles loaded with garlic essential oil : a new alternative to tebuconazole as seed dressing agent. Carbohydr. Polym..

[14] Liu X., Li T., Cui X., Tao R., Gao Z. (2024). Antifungal mechanism of nanosilver biosynthesized with Trichoderma longibrachiatum and its potential to control muskmelon Fusarium wilt. Sci. Rep..

[15] Krumova E. (2024). Exploring the mechanism underlying the antifungal activity of chitosan-based ZnO, CuO, and SiO2 nanocomposites as nanopesticides against Fusarium solani and Alternaria solani. Int. J. Biol. Macromol..

[16] Szopińska D. (2014).

[17] García-Simarro M.P. (2025). Two novel eco-friendly seed dressing nano-antifungals based on pH-responsive dendritic silica nanoparticles coated with chitosan and polydopamine. J. Environ. Chem. Eng..

[18] Boukaew S., Prasertsan P., Sattayasamitsathit S. (2017). Evaluation of antifungal activity of essential oils against aflatoxigenic Aspergillus flavus and their allelopathic activity from fumigation to protect maize seeds during storage. Ind. Crops Prod..

[19] Asimovic Z., Sarajevo F.S., Cengic L., Murtic S. (2016). Spectrophotometric determination of total chlorophyll content in fresh vegetables. Work. Fac. Agric. Food Sci. Unversity Sarajev..

[20] Lin J., Tang C. (2007). Determination of total phenolic and flavonoid contents in selected fruits and vegetables , as well as their stimulatory effects on mouse splenocyte proliferation. Food Chem..

[21] Kopec R.E., Cooperstone J.L., Cichon M.J., Schwartz S.J. (February. 2012).

[22] Yu Y.J. (2014). Facile synthesis of size controllable dendritic mesoporous silica nanoparticles. ACS Appl. Mater. Interfaces.

[23] Wang J., Sugawara-Narutaki A., Shimojima A., Osada M., Ma R., Okubo T. (2015). Dendritic silica nanoparticles synthesized by a block copolymer-directed seed-regrowth approach. Langmuir.

[24] Bhatia S. (2016).

[25] Filippov S.K. (2023). Dynamic light scattering and transmission electron microscopy in drug delivery: a roadmap for correct characterization of nanoparticles and interpretation of results. Mater. Horiz..

[26] Ma Y. (2023). pH-Mediated mucus penetration of Zwitterionic polydopamine-modified silica nanoparticles. Nano Lett..

[27] Ahuja U., Wang B., Hu P., Rethore J., Aifantis K.E. (2021). Polydopamine coated Si nanoparticles allow for improved mechanical and electrochemical stability. Electrochim. Acta.

[28] Xia Z. (2013). Facile synthesis of polydopamine-coated molecularly imprinted silica nanoparticles for protein recognition and separation. Biosens. Bioelectron..

[29] Deng C. (2021). Engineering of dendritic mesoporous silica nanoparticles for efficient delivery of water-insoluble paclitaxel in cancer therapy. J. Colloid Interface Sci..

[30] Hemmatpour H. (2023). New insights in polydopamine formation via surface adsorption. Nat. Commun..

[31] Sattary M., Amini J., Hallaj R. (2020). Antifungal activity of the lemongrass and clove oil encapsulated in mesoporous silica nanoparticles against wheat's take-all disease. Pestic. Biochem. Physiol..

[32] Vidyagauri V.L., Prabhanand Dalvi Uttam (2023). Application of induced bathochromic shift for trace level quantification of formaldehyde in some antiviral and anti-microbial drugs with cinnamaldehyde as 2,4-Dinitrophenyl hydrazine scavenger using UV-Visible spectroscopy. J. Anal. Chem..

[33] Cox H.J., Li J., Saini P., Paterson J.R., Sharples G.J., Badyal J.P.S. (2021). Bioinspired and eco-friendly high efficacy cinnamaldehyde antibacterial surfaces. J. Mater. Chem. B.

[34] Adam A. (2021). Orienting the pore morphology of core-shell magnetic mesoporous silica with the sol-gel temperature. Influence on MRI and magnetic hyperthermia properties. Molecules.

[35] Zahiri M., Babaei M., Abnous K., Taghdisi S.M., Ramezani M., Alibolandi M. (2020). Hybrid nanoreservoirs based on dextran-capped dendritic mesoporous silica nanoparticles for CD133-targeted drug delivery. J. Cell. Physiol..

[36] Wang Y., Song H., Yang Y., Liu Y., Tang J., Yu C. (2018). Kinetically controlled dendritic mesoporous silica nanoparticles: from Dahlia- to pomegranate-like structures by Micelle filling. Chem. Mater..

[37] Thommes M. (2015). Physisorption of gases, with special reference to the evaluation of surface area and pore size distribution (IUPAC technical report). Pure Appl. Chem..

[38] Yang X. (2022). Particle size and pore adjustment of dendritic mesoporous silica using different long alkyl-chain imidazolium ionic liquids as templates. Microporous Mesoporous Mater..

[39] Hadidi M., Pouramin S., Adinepour F., Haghani S., Jafari S.M. (2020). Chitosan nanoparticles loaded with clove essential oil: characterization, antioxidant and antibacterial activities. Carbohydr. Polym..

[40] Sattar S. (2022). Comparative analysis of microbial consortiums and nanoparticles for rehabilitating petroleum waste contaminated soils. Molecules.

[41] Zhang Y. (2025). Dual recombinase polymerase amplification system combined with lateral flow immunoassay for simultaneous detection of Staphylococcus aureus and Vibrio parahaemolyticus. J. Pharm. Biomed. Anal..

[42] Usman M.R. (2021). Degradation of ciprofloxacin by titanium dioxide (TiO2) nanoparticles: optimization of conditions, toxicity, and degradation pathway. Bull. Chem. React. Eng. Catal..

[43] Liu M. (2022). Silica nanostructures against fungal growth: design and preparation of antifungal cotton fabrics. Cellulose.

[44] Capeletti L.B. (2014). Tailored silica-antibiotic nanoparticles: overcoming bacterial resistance with low cytotoxicity. Langmuir.

[45] Derbalah A., Shenashen M., Hamza A., Mohamed A., El Safty S. (2018). Antifungal activity of fabricated mesoporous silica nanoparticles against early blight of tomato. Egypt. J. Basic Appl. Sci..

[46] Pariona N., Mtz-Enriquez A.I., Sánchez-Rangel D., Carrión G., Paraguay-Delgado F., Rosas-Saito G. (2019). Green-synthesized copper nanoparticles as a potential antifungal against plant pathogens. RSC Adv..

[47] Huang T., Li X., Maier M., O'Brien-Simpson N.M., Heath D.E., O'Connor A.J. (2023). Using inorganic nanoparticles to fight fungal infections in the antimicrobial resistant era. Acta Biomater..

[48] Ramya D., Sujatha P., Raghavendra K., Keshavulu K., Ramesh T., Radhika K. (2024). Antioxidants and polymer coating for soybean [Glycine max (L.) Merr.] seed longevity enhancement. Ind. Crops Prod..

[49] Yu C. (2020). Characteristics and hazards of the cinnamaldehyde oxidation process. RSC Adv..

[50] Nowak M., Tryniszewski W., Sarniak A., Wlodarczyk A., Nowak P.J., Nowak D. (2022). Concentration dependence of anti-and pro-oxidant activity of polyphenols as evaluated with a light-emitting Fe2+-Egta-H2 O2 system. Molecules.

[51] Hosseini S.F., Ghaderi J., Gómez-Guillén M.C. (2022). Tailoring physico-mechanical and antimicrobial/antioxidant properties of biopolymeric films by cinnamaldehyde-loaded chitosan nanoparticles and their application in packaging of fresh rainbow trout fillets. Food Hydrocoll..

[52] Li L., Pan X.L., Mu G.J. (2020). Toxic effects of potassium permanganate on photosystem II activity of Cyanobacteria microcystis aeruginosa.

[53] Goutam C., Bajpai B. (2019). Effect of KMnO 4 on Seed- borne fungi of Brassica campestris (Mustard).

[54] Kurek K., Plitta-michalak B., Ratajczak E. (2019).

[55] Yao Q., Zheng X., Zhou G., Zhang J. (2023). SGR-YOLO: a method for detecting seed germination rate in wild rice. Front. Plant Sci..

[56] Kardava K., Tetz V., Vecherkovskaya M., Tetz G. (2023). Seed dressing with M451 promotes seedling growth in wheat and reduces root phytopathogenic fungi without affecting endophytes. Front. Plant Sci..

[57] Kaur N., Sehgal S.K., Glover K.D., Byamukam E., Ali S. (2020). Impact of Fusarium graminearum on seed germination and seedling blight in hard red spring cultivars in South Dakota. J. Plant Pathol. Microbiol..

[58] Mondéjar-lópez M., Martínez J.C.G., Gómez-gómez L., Ahrazem O., Niza E. (2024). New gel from a water-soluble carboxymethyl chitosan-Cinnamaldehyde Schiff base Schiff base derivative as an effective preservative against soft rot in ginger. Food Chem..

[59] Dickinson A.J. (2019). β-Cyclocitral is a conserved root growth regulator. Proc. Natl. Acad. Sci. USA.

[60] Keke L. (2025). Silica nanoparticles enhanced seed germination and seedling growth of drought-stressed wheat by modulating antioxidant enzymes and mitigating lipid peroxidation. Environ. Sci. Nano.

[61] Chakraborti S., Bera K., Sadhukhan S., Dutta P. (2022). Bio-priming of seeds: plant stress management and its underlying cellular, biochemical and molecular mechanisms. Plant Stress.

[62] Zhao L. (2020). Nano-biotechnology in agriculture: use of nanomaterials to promote plant growth and stress tolerance. J. Agric. Food Chem..

[63] Chen J. (2021). Tebuconazole resistance of Fusarium graminearum field populations from wheat in Henan Province. J. Phytopathol..

[64] Chand Mali S., Raj S., Trivedi R. (2020). Nanotechnology a novel approach to enhance crop productivity. Biochem. Biophys. Reports.

[65] Mondéjar-López M., López-Jiménez A.J., Gómez-Gómez L., Ahrazem O., García-Martínez J.C., Niza E. (2024). Field crop evaluation of polymeric nanoparticles of garlic extract–chitosan as biostimulant seed nano-priming in cereals and transcriptomic insights. Polymers.

[66] Ferguson L.R. (2001). Role of plant polyphenols in genomic stability. Mutat. Res. Fundam. Mol. Mech. Mutagen..

[67] Šamec D., Karalija E., Šola I., Vujčić Bok V., Salopek-Sondi B. (2021). The role of polyphenols in abiotic stress response: the influence of molecular structure. Plants.

[68] Stra A., Almarwaey L.O., Alagoz Y., Moreno J.C., Al-Babili S. (2023). Carotenoid metabolism: new insights and synthetic approaches. Front. Plant Sci..

[69] Kretschmer M., Damoo D., Djamei A., Kronstad J. (2020). Chloroplasts and plant immunity: where are the fungal effectors?. Pathogens.

[70] Ebrahimi P., Shokramraji Z., Tavakkoli S., Mihaylova D., Lante A. (2023). Chlorophylls as natural bioactive compounds existing in food by-products: a critical review. Plants.

[71] Jaime C., Muchut S.E., Reutemann A.G., Gieco J.O., Dunger G. (2020). Morphological changes, alteration of photosynthetic parameters and chlorophyll production induced by infection with alfalfa dwarf virus in Medicago sativa plants. Plant Pathol..

[72] Al-Khayri J.M. (2023). The role of nanoparticles in response of plants to abiotic stress at physiological, biochemical, and molecular levels. Plants.

[73] (EC) No 1107/2009, (EC) No 1107/2009 http://eur-lex.europa.eu/LexUriServ/LexUriServ.do?uri=OJ:L:2009:309:0001:0050:en:PDF.

[74] Hussain M.I. (2022). Benzoxazinoids in wheat allelopathy – from discovery to application for sustainable weed management. Environ. Exp. Bot..

[75] Larras F. (2022). A meta-analysis of ecotoxicological models used for plant protection product risk assessment before their placing on the market. Sci. Total Environ..

[76] Ratier A., Lopes C., Multari G., Mazerolles V., Carpentier P., Charles S. (2022). New perspectives on the calculation of bioaccumulation metrics for active substances in living organisms. Integrated Environ. Assess. Manag..

[77] Scott J.G., Buchon N. (2019). Drosophila melanogaster as a powerful tool for studying insect toxicology. Pestic. Biochem. Physiol..

[78] Kissoum N., Bensafi-Gheraibia H., Hamida Z.C., Soltani N. (2020). Evaluation of the pesticide Oberon on a model organism Drosophila melanogaster via topical toxicity test on biochemical and reproductive parameters. Comp. Biochem. Physiol., Part C: Toxicol. Pharmacol..

